# Relationship between hypertension during dialysis and body composition in non-overweight/overweight obese patients

**DOI:** 10.1371/journal.pone.0333566

**Published:** 2026-04-21

**Authors:** Jiaoyan Chen, Xianqiong Lu, Jurong Yang, Jingrong Peng, Maodi She, Yunyan Wang

**Affiliations:** Department of Nephrology, The Third Affiliated Hospital of Chongqing Medical University (FangDa Hospital), Chongqing, China; University of Pisa, ITALY

## Abstract

Body composition parameters (such as BMI and waist-to-hip ratio) have a certain predictive value for blood pressure. In patients undergoing maintenance hemodialysis (MHD), BMI can affect dialysis adequacy and blood pressure control levels, and there are differences in body composition at different BMI levels. The aim of this study was to investigate the association between hypertension during dialysis and body composition in non-overweight/overweight obese patients. A total of 248 patients undergoing maintenance hemodialysis (MHD) at this center were enrolled. Body composition was measured using an InBody bioelectrical impedance analyzer prior to dialysis. Intra-dialysis blood pressure data from the preceding three months were collected via the hemodialysis system. Patients were categorized based on their dry weight at enrollment: those with BMI < 23 kg/m² were classified as the non-overweight group, and those with BMI ≥ 23 kg/m² as the overweight/obese group. LASSO regression was employed to identify body composition variables associated with hypertension during hemodialysis. Based on LASSO regression results, multivariate linear regression and logistic regression analyses were conducted to evaluate the impact of pre-dialysis body composition on blood pressure (BP) in non-overweight and overweight/obese hemodialysis patients. The overall prevalence of hypertension was 86% (214/248), and the proportion of overweight/obese patients was 41.0% (102/248). TBW and Protein were positively correlated with hypertension in non-overweight male patients (OR (95% CI): 1.28 (0.34–1.98); OR (95% CI): 1.97 (1.51–3.33)), and BMC was negatively associated with hypertension in non-overweight male patients (OR (95% CI): 0.10 (0.01–0.70)). In overweight obese female patients, Fat was positively associated with hypertension (OR (95% CI): 1.12 (1.01–2.26)). This study identified risk factors for elevated blood pressure associated with gender and BMI in a subset of the MHD Asian population. The study also provided evidence that different body composition factors (such as total body water, protein, bone mineral content, and fat) drive hypertension risk in different MHD subgroups, rather than being determined solely by BMI.

## Introduction

Hypertension is prevalent in end-stage renal disease (ESRD) patients on hemodialysis, with prevalence rates reported to be as high as 90% in patients on maintenance hemodialysis (MHD) [1,2]. Growing evidence suggests that hypertension in dialysis increases the risk of death and cardiovascular events in patients with MHD [3]. The pathogenesis of hypertension in patients with MHD is complex and manifests itself as an interaction of extracellular hypervolemia, overactive sympathetic and renin-angiotensin-aldosterone systems, vascular pathology, obesity, advanced age, and other clinical conditions [4], which also significantly increases the difficulty of managing and controlling blood pressure in MHD patients.

The number of overweight and obese patients with chronic kidney disease (CKD) is increasing. Obesity is not only a recognized risk factor for hypertension [5], it is also one of the most common causes of CKD [6]. Body mass index (BMI) affects dialysis adequacy and blood pressure control in hemodialysis (HD) patients [7]. Hsu et al [8] noted that patients who are overweight or obese have more than three times the risk of developing end-stage renal disease (ESRD) compared to patients with ideal body weight. Obesity can directly contribute to CKD as an independent risk factor [9]. The effect of obesity on CKD may involve activation of the renin-angiotensin-aldosterone system (RAAS) and the interaction of leptin, lipocalin, fetuin-A, and adipose tissue [10]. Studies have shown that increased BMI is associated with increased expression of pro-inflammatory cytokines, increased oxidative stress load, and adipocytokine imbalance in patients with MHD [11], but there is still debate about the impact of obesity on CKD progression in patients with MHD [12,13].

Body composition monitoring is an important tool to assess dry weight goals in MHD patients and to better control the occurrence of dialysis events (e.g., hypotension, headache, etc.) [14]. Body composition parameters (e.g., BMI, waist-to-hip ratio) have some predictive value for blood pressure [15]. Korhonen et al [16] showed that adult adiposity, lean body mass, and blood pressure are positively correlated and that higher muscle mass may increase the risk of cardiovascular disease. The combination of a low lean body mass index (LTI) and a high adipose tissue index (FTI) may increase the risk of cardiovascular hospitalization in patients with MHD [17]. While Tian et al [18] reported that low LTI and high FTI were associated with hypotension (IDH) in dialysis. In addition, body composition varies across BMI levels. Prunceva et al [19] reported that skeletal muscle mass (SMM) and basal metabolic rate (BMR) are also higher in those with higher BMI.

Currently, reports on the relationship between BMI and blood pressure in MHD patients are inconsistent, and research on the relationship between body composition and hypertension in MHD patients is limited. It is necessary to gain a clearer understanding of the association between body composition and hypertension in MHD patients and the value of BMI in this context, as this could provide a theoretical framework for better clinical management of hypertension in MHD patients.

## Materials and methods

### Participants

Participants in this study were dialysis patients from the dialysis center of a tertiary general hospital in Chongqing, and the inclusion criteria were (1) age ≥ 18 years. (2) The duration of maintenance hemodialysis was ≥ 3 months, with an average of 2–3 times per week, 3.5–4 h/session. (3) No acute intercurrent illnesses had occurred in the 2 weeks before enrollment, and their clinical condition was relatively stable. (4) Informed consent was obtained. The exclusion criteria were: (1) those who combined with severe heart valve disease, atrial fibrillation, acute myocardial infarction, severe heart failure, cerebral infarction, severe edema, and severe infection. (2) Those with mental abnormalities, cognitive disorders, and those who are unable to take care of themselves. (3) Significant changes in dry weight or poor blood pressure control within 3 months prior to enrollment. (4) History of diabetes, malignant tumors, or other comorbidities associated with poor prognosis. The study protocol was adopted and approved by the hospital ethics committee. During the screening process, a total of 66 patients were excluded due to meeting exclusion criteria, primarily including poor blood pressure control within 3 months prior to enrollment (3 cases), malignant tumors (2 cases), and a history of diabetes (61 cases).

Participants were recruited through research projects. Prior to project participation, participants signed written informed consent forms; the project was approved by the Research Ethics Committee of Chongqing Medical University Third Affiliated Hospital (No. 20240085). The recruitment period for body composition measurements spanned from August 1, 2024, to November 1, 2024. Blood pressure data were extracted from the center’s information system during the same period (August 1 to November 1, 2024), collected and recorded anonymously.

### Body composition measurement

Body composition at enrollment was measured using a mobile whole-body bioelectrical impedance instrument (InBody S10). Measurements were performed approximately 30 minutes before hemodialysis by a trained dialysis nurse and a nephrologist. Measurements were made by placing electrodes on the patient’s thumbs and middle fingers of both upper limbs and to the calf side of both lower limbs while the MHD patient was in the supine position. Final measurements were collected: Total Body Water (TBW), Protein, Bone Mineral Content (BMC), Fat, Basal Metabolic Rate (BMR), Mineral, Fat Free Mass (FFM), Visceral Fat Area (VFA), Muscle Mass (SLM), Skeletal Muscle Mass (SMM), Percent Body Fat (PBF)

### Blood pressure data collection and definition of hypertension

Blood pressure data for MHD patients is collected through this center’s hemodialysis information system. Each blood pressure reading is measured and recorded by two professionally trained hemodialysis nurses. We use the average blood pressure during dialysis sessions occurring within approximately three months prior to the enrollment date as the final blood pressure for MHD patients. Currently, there is uncertainty about the optimal blood pressure level and type of measurement in MHD patients. Although home and ambulatory blood pressure monitoring are superior, with home blood pressure values and ambulatory blood pressure measurements correlating more strongly with left ventricular hypertrophy as well as cardiovascular mortality than clinically measured blood pressure [20,21]. However, adherence to ambulatory and home blood pressure measurements is considered a barrier for most patients due to practical limitations [22]. Our aim was to assess safety and feasibility information for this trial to determine the optimal blood pressure value during dialysis. There is also much evidence on the predictive effect of blood pressure values in the dialysis unit [22]. The criteria for determining hypertension were based on the guidelines for the diagnosis and treatment of cardiovascular disease in patients with chronic kidney disease (dialysis) and were combined with the blood pressure judgment of patients with MHD: systolic blood pressure≥140 mmHg and/or diastolic blood pressure≥90 mmHg [23].

### Definition of non-overweight/overweight obese

We applied the World Health Organization’s Asian population standard [24] and based on the dry weight of the patients at the time of enrollment, we defined the non-overweight group as having a BMI < 23 kg/m² and the overweight/obese group as having a BMI ≥ 23 kg/m².

### Statistical analysis

Descriptive statistics were calculated for continuous and categorical variables. Normally distributed continuous variables were expressed as mean ± standard deviation. Quantitative variables were described as mean±standard deviation, and qualitative variables were described as ratios or proportions (%). Differences in quantitative variables between genders were analyzed using the t-test. The chi-square test was used to analyze the differences in qualitative variables between the two groups. Body composition variables associated with hypertension were screened by LASSO regression. LASSO analysis converged regression coefficients to zero through a penalizing Lambda coefficient, thereby distinguishing excluded variables with zero coefficients from selected variables with non-zero coefficients to prevent overfitting and multicollinearity. These selected variables include TBW, Protein, BMC, Fat, which are considered to be most associated with hypertension. It is possible that the potential relationship between BMI and Fat resulted in the exclusion of BMI after the increase of the penalty term. To further validate our previously set ideas, after adjusting for age, we proceeded to analyze the relationship of these selected variables with blood pressure at different levels of BMI using multivariate linear regression. The effect of selected variables on hypertension at different BMI levels was analyzed by multivariate logistic regression. *P* < 0.05 was statistically significant.

## Result

**Fig 1:**
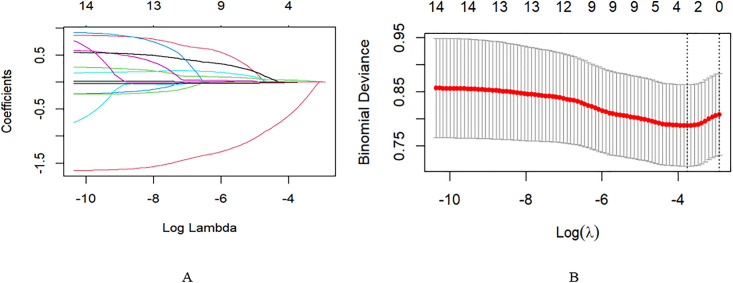
Process of screening variables by LASSO regression modeling. (A)LASSO coefficients for a total of 14 indicators. (B)log(λ) and biased likelihood variance. Vertical dashed lines are plotted according to the minimum criterion and the 1 SE of the minimum criterion (the 1-SE criterion).

We defined hypertension as a dichotomous dependent variable (Yes = 1, No = 0) and included 14 initial variables in the LASSO regression model for feature selection, including Total Body Water (TBW), Protein, Bone Mineral Content (BMC), Fat, Basal Metabolic Rate (BMR), Mineral, Fat Free Mass (FFM), Visceral Fat Area (VFA), Muscle Mass (SLM), Skeletal Muscle Mass (SMM), Percent Body Fat (PBF), Age, Sex, and BMI. Fig 1A, coefficients for a total of 14 metrics. The 10-fold cross-validation method was applied to the iterative analysis. Because the penalty term increased, 4 variables were retained in the model as λ approached 0.02337, including TBW, Protein, BMC, and Fat. (Fig 1B).

We characterize the selected variables associated with hypertension as described above, and in addition to this, although age was excluded due to the increase in the penalty term, we still included age because it is widely considered to be associated with blood pressure and could be a potential confounder. As shown in Table 1, a total of 248 MHD patients with hypertension were 86% (214/248), 88.7% (118/133) in men and 83.5% (96/115) in women. The prevalence of overweight obesity was 41.0% (102/248), 39.8% (53/133) in men and 42.6% (49/115) in women. SBP was higher in males than females *(P* < 0.05), and TBW, Protein, and BMC were higher in males than females (*P* < 0.05).

**Table 1:** Comparison of patient characteristics between male and female MHD patients.

Variables	Male (n = 133)	Female(n = 115)	P
SBP	159.6 ± 19.1	153.1 ± 20.5	0.012
DBP	84.5 ± 13.2	82.3 ± 13.1	0.385
TBW	38.46 ± 6.34	29.09 ± 4.39	*<*0.001
Protein	10.06 ± 1.70	7.59 ± 1.19	*<*0.001
BMC	3.36 ± 0.69	2.67 ± 0.47	*<*0.001
Fat	15.55 ± 8.62	14.83 ± 8.27	0.500
Age	57.81 ± 14.89	57.96 ± 15.05	0.937
BMI	23.72 ± 4.35	22.91 ± 4.11	0.608
Hypertension			0.230
No	15(11.2)	19(16.5)	
Yes	118(88.7)	96(83.5)	
Overweight andobese			0.730
No	80(60.2)	66(57.4)	
Yes	53(39.8)	49(42.6)	

SBP, systolic blood pressure; DBP, diastolic blood pressure; TBW, total body water; BMC, bone mineral content; BMI, body mass index.

After adjusting for age, multiple linear regression was performed with systolic blood pressure as the dependent variable and TBW, Protein, BMC, and Fat as independent variables. In non-overweight obese male patients, TBW and Protein were positively correlated with SBP(*β*(SE)=0.25(0.40), *P* = 0.033; *β*(SE)=0.21(1.50), *P* = 0.029), and BMC was negatively correlated with SBP(*β*(SE)=−0.20(1.30), *P* = 0.048). In overweight obese female patients, Fat was positively correlated with SBP (*β*(SE)=0.23(1.40), *P* = 0.015). See Table 2 for details.

**Table 2:** Relationship of SBP with TBW, Protein, BMC, and Fat.

Independent variables		Beta	SE	t	P	95% CI	
SBP in Male							
Non-overweight obese	TBW	0.245	0.404	2.163	0.033	0.071	2.679
	Protein	0.213	1.510	1.835	0.029	0.132	5.771
	BMC	−0.197	1.300	−1.807	0.048	−6.524	1.599
	Fat	0.102	0.240	0.926	0.357	0.255	1.698
	Age	0.095	0.128	0.889	0.376	−0.140	1.968
Overweight and obese	TBW	0.104	0.572	0.651	0.518	−0.782	1.527
	Protein	0.107	1.228	0.654	0.516	−3.038	5.954
	BMC	−0.056	1.514	−0.334	0.740	−8.967	4.188
	Fat	0.010	0.367	0.068	0.946	0.715	0.765
	Age	−0.059	0.249	−0.386	0.701	−0.406	0.598
SBP in Female							
Non-overweight obese	TBW	0.088	0.791	0.703	0.484	−1.022	2.135
	Protein	0.109	2.143	0.832	0.408	−3.421	8.32
	BMC	−0.046	1.349	−0.358	0.721	−8.388	7.936
	Fat	0.022	0.279	0.180	0.858	0.506	0.606
	Age	0.054	0.165	0.459	0.647	−0.253	0.505
Overweight and obese	TBW	0.123	0.675	0.624	0.536	−0.945	1.788
	Protein	0.140	2.061	0.641	0.525	−3.953	7.622
	BMC	0.009	1.462	0.046	0.964	−2.773	3.367
	Fat	0.227	1.383	1.415	0.015	0.023	4.318
	Age	−0.091	0.175	−0.579	0.566	−0.855	−0.003

SBP, systolic blood pressure; TBW, total body water; BMC, bone mineral content.

After adjusting for age, multiple linear regression was performed with diastolic blood pressure as the dependent variable and TBW, Protein, BMC, and Fat as independent variables. As shown in Table 3, in non-overweight obese male patients, TBW was positively correlated with DBP (*β*(SE)=0.17 (1.27), *P* = 0.013) and BMC was negatively correlated with DBP (*β*(SE = −0.183(2.25), *P* = 0.034).

**Table 3:** Relationship of DBP with TBW, Protein, BMC, and Fat.

Independent variables		Beta		SE	t	P	95% CI
DBP in Male							
Non-overweight obese	TBW	0.170	1.267	1.601	0.013	0.103	2.957
	Protein	0.063	1.044	0.548	0.585	−1.503	2.647
	BMC	−0.183	2.249	−1.725	0.034	−2.592	1.349
	Fat	0.020	0.165	0.184	0.854	−0.358	0.297
	Age	−0.271	0.087	−2.612	0.081	−3.054	0.399
Overweight and obese	TBW	0.172	0.357	1.142	0.206	0.313	1.129
	Protein	0.258	1.410	1.657	0.105	0.510	5.183
	BMC	0.189	1.276	1.262	0.214	2.474	6.739
	Fat	0.123	0.239	0.813	0.420	0.278	1.688
	Age	−0.221	0.162	−1.489	0.144	−2.386	−0.068
DBP in Female							
Non-overweight obese	TBW	0.138	0.479	1.176	0.244	−0.392	1.519
	Protein	0.220	1.670	1.905	0.131	−0.149	6.511
	BMC	−0.181	1.746	−1.548	0.126	−6.270	−1.669
	Fat	0.026	0.178	0.221	0.826	−0.315	0.394
	Age	−0.320	0.101	−2.842	0.126	−3.585	0.486
Overweight and obese	TBW	0.083	0.372	0.528	0.600	−0.556	0.949
	Protein	0.089	1.985	0.405	0.687	−3.211	4.821
	BMC	−0.010	1.603	−0.062	0.951	−7.507	7.058
	Fat	0.155	0.260	0.348	0.106	−0.616	0.435
	Age	−0.043	0.121	−0.270	0.788	−0.312	0.278

DBP, diastolic blood pressure; TBW, total body water; BMC, bone mineral content.

**Fig 2:**
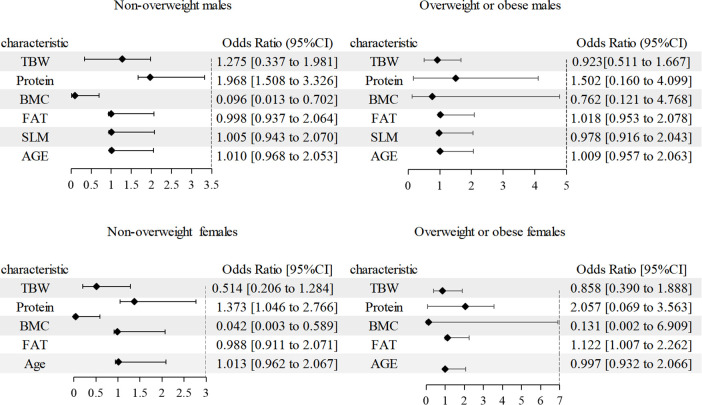
Association of TBW, Protein, BMC, and Fat with hypertension risk.

After adjusting for age, in binary logistic regression analysis with hypertension (Yes = 1, No = 0) as the dependent variable and TBW, Protein, BMC, and Fat as independent variables, TBW and Protein were positively associated with hypertension in non-overweight males(OR (95% CI): 1.28 (0.34–1.98), *P* = 0.012; OR (95% CI): 1.97 (1.51–3.33), *P* = 0.009), and BMC was negatively associated with hypertension in non-overweight males (OR (95% CI): 0.10 (0.01–0.70), *P* = 0.021). In overweight obese females, Fat was positively associated with hypertension (OR (95% CI): 1.12 (1.01–2.26), *P* = 0.036). Details were shown in the Fig 2.

## Discussion

This study identified gender- and BMI-specific body composition predictors associated with hypertension in patients with MHD, and we found that TBW and Protein were positively associated with hypertension in non-overweight male MHD patients, and BMC was negatively associated with hypertension in non-overweight male MHD patients. Fat was positively associated with hypertension in overweight obese female MHD patients.

CKD and progressive loss of kidney function can lead to sodium and water retention in the body, which can lead to arterial hypertension [25]. Currently, volume overload is the first problem to be addressed in the dialysis management of hypertension in HD patients [26]. Extracellular fluid overload and poor fluid management underlie the development of cardiovascular complications in HD patients [27]. Over time and with repeated positive fluid imbalances, patients with HD are susceptible to the accumulation of body sodium and water, which can lead to an adverse outcome of cardiovascular events from extracellular fluid overload [28]. Optimal management of fluid and sodium imbalances in HD patients can be achieved through salt intake restriction and fluid removal, dialysis and real-time monitoring and assessment of dry weight during dialysis [29]. Yet, restoring extracellular fluid homeostasis, achieving adequate blood pressure control, and maintaining ideal hemodynamic stability in dialysis patients remains a significant challenge [30]. With the support of new diagnostic and monitoring tools such as multi-frequency Bioimpedance Spectroscopy (BIS), Artificial Intelligence algorithms, etc., we will enter a new era of proposing more precise fluid management methods to ensure optimal fluid status control.

Our study found that blood pressure (BP) in non-obese MHD male patients is also associated with body protein content, and this relationship may be linked to more severe kidney function impairment. Chronic kidney damage is known to directly lead to elevated blood pressure, particularly in end-stage renal disease (ESRD) patients undergoing hemodialysis [31]. Protein-energy wasting is common in CKD patients [32], and more severe kidney function impairment may also result in greater protein-energy wasting [33], which may provide a reasonable explanation for our findings. However, whether there is a direct underlying association between BP and body protein content requires further investigation.

Previous studies have reported an association between hypertension and bone mineral loss [34], The relationship between hypertension and bone mineral content may be through a pathophysiologic link between blood pressure regulation and calcium metabolism [35], Persistent hypercalciuria and hyperparathyroidism as pathophysiologic mechanisms of increased bone mineral loss in hypertensive patients [36]. This supports our findings to some extent. Calcium-phosphorus metabolism disorders and hypertension are prevalent in MHD patients, and mineral and bone disorders (CKD-MBD) increase the risk of adverse outcomes, including cardiovascular events, in hemodialysis (HD) patients [37], monitoring bone mineral content may aid in the therapeutic management of MHD patients. In future studies, we will further incorporate laboratory indicators such as serum calcium, serum phosphorus, and parathyroid hormone to comprehensively explore the relationship between mineral metabolism and blood pressure.

In this study, we did not find any relationship between TBW and Protein and blood pressure in overweight and obese patients, which warrants further reflection on the impact of overweight and obesity on MHD patients. Li et al [38] found a nearly threefold increased risk of cardiovascular death in peritoneal dialysis patients who were obese and had uncontrolled blood pressure compared to normal weight. Obesity and hypertension are closely related, and adiposity increases the risk of insulin resistance, hypertension, and cardiovascular disease [39]. In addition, there is a “reverse epidemiology” hypothesis regarding the relationship between body mass index (BMI) and blood pressure (BP) in HD patients, with obese dialysis patients having a longer survival time [40,41]. Hou et al [42] found that overweight/obesity played a major mediating role in the correlation between lifestyle risk factors and systolic and diastolic blood pressure, as well as being at the top of the list of modifiable risk factors for hypertension. The interaction of BMI may also have had some impact on our findings. For example, Xu et al [43] found that the relationship between heart rate and the incidence of type 2 diabetes mellitus in a rural Chinese population was particularly pronounced in non-overweight/obese participants compared to overweight and obese individuals, which was due to the interaction between heart rate and BMI. However, we only found an association between Fat and BP in overweight/obese female patients, but not in males, which may be influenced by sex differences in blood pressure hemodynamics in overweight/obese individuals [44]. The gender-specific mechanisms underlying fat distribution patterns, sex hormone levels, and insulin resistance may account for sex-related differences. Sironi [45] et al. noted that visceral fat is closely associated with hypertension, insulin resistance, and metabolic syndrome. Postmenopausal women experience increased visceral fat accumulation due to declining estrogen levels, making adipose tissue a primary driver of hypertension. In male patients of this study, the association between TBW and Protein with hypertension may reflect more severe metabolic disorders and a state of protein-energy depletion. Conversely, the role of Fat in female patients more directly highlights the core position of adipose tissue as an endocrine organ in blood pressure regulation.

It is important to note that “reverse epidemiology” primarily describes the paradoxical association between obesity and mortality, and does not imply that obesity negates the risk of hypertension. This study found that in overweight and obese female patients, body fat content was independently and positively correlated with hypertension risk. This suggests that in the dialysis population, obesity may simultaneously impact survival prognosis and blood pressure regulation through distinct pathophysiological pathways. Therefore, in clinical practice, even for patients who may derive survival benefits from a higher body mass index, identifying and managing hypertension driven by specific body composition factors—such as excessive body fat—remains a critical component of optimizing cardiovascular health management. In non-overweight men, monitoring total body water (TBW) and protein status aids in identifying hypertension risk; in overweight/obese women, controlling body fat may be more beneficial for blood pressure management. This suggests that individualized intervention strategies should be developed based on body composition rather than relying solely on BMI.

We acknowledge several limitations of this study. First, similar to other cross-sectional studies, unaccounted variables or inadequately adjusted factors may introduce confounding effects. For example, due to data limitations, we were unable to include residual renal function, blood pressure variability, ultrafiltration volume, antihypertensive medication use, and other parameters in the analysis. Given that antihypertensive medication use and blood pressure variability may influence study outcomes, we conducted a three-month follow-up of patients’ blood pressure during dialysis sessions. This extended assessment may more comprehensively reflect patients’ true dynamic blood pressure patterns, thereby mitigating the impact of medications and blood pressure fluctuations on results. Furthermore, the inclusion of total body water (TBW) provides robust indirect evidence and physiological relevance supporting the clinical significance of ultrafiltration volume. Additionally, we lack documentation regarding the menopausal status and estrogen use among female patients, factors that may influence blood pressure regulation and thus constitute potential confounders when interpreting gender-related differences. Future studies should prospectively integrate these therapeutic parameters with body composition metrics to establish a complete evidence chain linking “therapeutic interventions” to “physiological states” and ultimately to “clinical outcomes”, thereby providing a foundation for precision management. Second, this study excluded patients with diabetes to control for the disease’s strong confounding effects on blood pressure and fluid balance. However, diabetes is a significant comorbidity among patients with chronic kidney disease and those on maintenance hemodialysis, often associated with more pronounced volume overload, metabolic disturbances, and blood pressure variability. Therefore, the findings of this study apply only to non-diabetic populations. Future research should validate the relationship between body composition and hypertension in diabetic patients to provide more universally applicable clinical guidance. Finally, individual variations may influence body composition and its association with outcomes, necessitating caution when extrapolating the findings of this study.

## Conclusions

We found that the relationship between body composition and BP in MHD patients differed across BMI levels and gender. In non-overweight male patients, TBW and Protein had a positive effect on BP, whereas BMC had a negative effect on hypertension. In overweight obese female patients, Fat had a positive effect on hypertension. These findings help us to obtain more useful information on hypertension management from the body composition data of MHD patients and provide some references for MHD patients during clinical treatment.

## Supporting information

S1 File Raw data. (CSV)
