# Supplementary material for: Relationship between hypertension during dialysis and body composition in non-overweight/overweight obese patients
Source: PLoS One. 2026 Apr 21;21(4):e0333566. doi: 10.1371/journal.pone.0333566 (PMC13098967; doi:10.1371/journal.pone.0333566)
Supplement: Attachment — Submitted filename: Response to Reviewers.docx [file pone.0333566.s003.docx]

**Dear editor:**

Thank you for your letter comments on our manuscript entitled “Relationship between Hypertension During Dialysis and Body Composition in Non-Overweight/Overweight Obese Patients” (EMI:56c0be9ce8402b9a). Those comments are very helpful for revising and improving our paper, we have carefully studied these comments and made corrections. In the following pages are our point-by-point responses to each of the comments.

**Reviewers' comments:**

**Reviewer's Responses to Questions**

**Comments to the Author**

**1. Is the manuscript technically sound, and do the data support the conclusions?**

The manuscript must describe a technically sound piece of scientific research with data that supports the conclusions. Experiments must have been conducted rigorously, with appropriate controls, replication, and sample sizes. The conclusions must be drawn appropriately based on the data presented.

Reviewer #1: Yes

Reviewer #2: No

**2. Has the statistical analysis been performed appropriately and rigorously?**

Reviewer #1: Yes

Reviewer #2: No

**3. Have the authors made all data underlying the findings in their manuscript fully available?**

The PLOS Data policy requires authors to make all data underlying the findings described in their manuscript fully available without restriction, with rare exception (please refer to the Data Availability Statement in the manuscript PDF file). The data should be provided as part of the manuscript or its supporting information, or deposited to a public repository. For example, in addition to summary statistics, the data points behind means, medians and variance measures should be available. If there are restrictions on publicly sharing data—e.g. participant privacy or use of data from a third party—those must be specified.

Reviewer #1: Yes

Reviewer #2: Yes

**4. Is the manuscript presented in an intelligible fashion and written in standard English?**

PLOS ONE does not copyedit accepted manuscripts, so the language in submitted articles must be clear, correct, and unambiguous. Any typographical or grammatical errors should be corrected at revision, so please note any specific errors here.

Reviewer #1: Yes

Reviewer #2: No

**5. Review Comments to the Author**

Please use the space provided to explain your answers to the questions above. You may also include additional comments for the author, including concerns about dual publication, research ethics, or publication ethics. (Please upload your review as an attachment if it exceeds 20,000 characters)

**Reviewer #1:**

**Dear authors, congratulations on the manuscript and interest in the subject of hypertension in dialysis. I have a few comments I'd like to make about the work:**

**1) At what point during the hemodialysis session was the blood pressure value captured for the diagnosis of arterial hypertension?**

**Response:** Thanks for your valuable comments. The blood pressure data used in this study were derived from the average blood pressure recorded during dialysis sessions in the dialysis center information system, specifically the average blood pressure values from each dialysis session within the three months preceding enrollment. Blood pressure during each dialysis session was measured and recorded by two professional nurses. We did not distinguish specific time periods (pre-, intra-, or post-dialysis) but instead used the overall average value throughout the entire dialysis session as the basis for analysis. This approach partially reflects the overall blood pressure burden during the dialysis process. In subsequent studies, we may further refine the time periods to analyze blood pressure heterogeneity.

**2) How many individuals were excluded from the study based on the exclusion criteria?**

**Response:** Thanks for your valuable comments. As you have considered, to further enhance the transparency and reproducibility of the study, we have supplemented the “Materials and Methods” section with details on the number of cases excluded during the screening process and their primary reasons.

**3) Why was LASSO regression used and not ELASTIC NET regression ?**

**Response:** Thanks for your valuable comments. LASSO regression is suitable for automatic variable selection in high-dimensional data, effectively preventing overfitting. It is appropriate for this study's initial scenario with a large number of variables (14). As you considered, Elastic Net combines the advantages of LASSO and ridge regression. However, when the sample size is moderate and multicollinearity among variables is not pronounced (as in this study), LASSO already performs adequate feature selection. Therefore, LASSO regression was selected.

4**) What about residual renal function? We know that loss of residual renal function compromises blood volume control and can impact blood pressure, while also contributing to changes in body composition. Could the men in this study, who had a correlation between total body water and systolic blood pressure, have lower residual renal function?**

**Response:** Thanks for your valuable comments and the question you raise is indeed critical. Residual kidney function (RKF) may indeed influence a patient's volume status and blood pressure levels by affecting the efficiency of sodium and water clearance. Although this study included only end-stage renal disease patients on regular dialysis for ≥3 months (most with severely impaired or absent RKF), inter-individual variations in residual renal function may still represent a potential confounding factor. This represents one limitation of our study, which we addressed in the Discussion section. Future research plans include incorporating more direct measures of RKF (such as urea clearance or urine output) to further elucidate its role in the relationship between body composition and blood pressure.

**5) The authors suggested that mineral and bone metabolism disorders could partially explain the correlation between bone mass composition parameters and arterial hypertension in men. However, in this study, this is merely speculative, since hypercalciuria appears to be negligible in dialysis patients, and the lack of laboratory data on mineral metabolism variables (parathyroid hormone, calcium, and phosphorus levels) precludes any conclusions in this regard. I agree, however, that new studies evaluating these laboratory variables should be carried out.**

**Response:** Thanks for your valuable comments and we fully agree with your perspective. The discussion regarding the association between bone mineral content and hypertension in the manuscript is indeed speculative. In future studies, we will incorporate additional laboratory indicators such as serum calcium, serum phosphorus, and parathyroid hormone to more comprehensively explore the relationship between mineral metabolism and blood pressure. We have revised the relevant discussion to ensure the rigor of our findings, as detailed at the end of the fourth paragraph in the Discussion section.

**6) The question of "reverse epidemiology" in patients receiving dialysis is quite interesting, but it links obesity and overweight to lower mortality, and does not necessarily include hypertension in this conclusion. I would like the authors to weigh in more assertively at this point, specifically in dialysis patients.**

**Response:** Thanks for your valuable comments. In the sixth paragraph under discussion, we supplement and emphasize that: although an “inverse epidemiological” phenomenon linking obesity to mortality may exist in the dialysis population, this does not negate obesity's role as a risk factor for hypertension. Our study provides new evidence for understanding the complex relationship between obesity and hypertension in dialysis patients from the perspective of body composition analysis. It further underscores that actively managing hypertension driven by factors such as body fat remains crucial in the clinical management of hemodialysis patients, even for those who may benefit from a higher BMI in terms of survival.

**7) Diabetic patients with chronic kidney disease have higher blood pressure values and are hypervolemic. Why was the variable diabetes mellitus not considered in this study?**

**Response:** Thanks for your valuable comments. As you have noted, diabetes is a common and significant comorbidity among patients on maintenance hemodialysis, with profound effects on blood pressure, volume load, and metabolic status. During the study design phase, to more clearly examine the direct association between body composition and hypertension while avoiding strong confounding effects from diabetes and its related complications (such as autonomic neuropathy and more complex volume issues), diabetic patients were deliberately excluded. Therefore, the conclusions of this study primarily apply to the non-diabetic MHD population. We have supplemented this point in the limitations section of the “Discussion” and emphasize that future research should validate these associations in diabetic populations to provide more universally applicable clinical guidance.

**Reviewer #2:**

**1) Thank you for the effort and work put into this study. Hypertension is highly prevalent among MHD patients and is influenced by multiple inter-related factors. To further strengthen the findings and interpretation, additional information would be helpful, such as blood pressure variability across the dialysis phases (pre-, intra- and post-haemodialysis), details of antihypertensive medications and compliant, ultrafiltration volume, and other dialysis-related parameters as mentioned. These variables are important to better justify and contextualise the associations with BIA parameters. Incorporating these, may enhance the clinical relevance of the study and provide deeper insights into how BIA findings can be applied to optimise blood pressure management in MHD patients.**

**Response:** Thanks for your valuable comments. We fully agree that parameters such as blood pressure variability, antihypertensive medication use, and ultrafiltration volume are crucial for gaining a deeper understanding of the relationship between body composition and blood pressure in the context of hemodialysis. Due to the retrospective design of this study, there were certain limitations in obtaining some parameters:

Antihypertensive Medications: Patients used a wide variety of antihypertensive drugs. As this was a retrospective analysis, systematic classification and dose adjustment analysis (e.g., specific types, doses, and medication adherence) were challenging. Therefore, these were not included as covariates in the model. We further acknowledge this as a limitation of the study.

Blood pressure variability: To minimize random error from single measurements, we used the average of all blood pressure readings taken during dialysis sessions within the three months prior to enrollment to reflect the patient's overall blood pressure burden. However, this approach cannot precisely quantify dynamic blood pressure changes during dialysis (e.g., blood pressure variability or intradialytic hypotension events). We have supplemented this point in the discussion as a direction for future research.

Ultrafiltration Volume: Your point is crucial. While this study did not directly include ultrafiltration volume in the model, one of its core findings—that total body water (TBW) is an independent positive predictor of hypertension in non-overweight male patients—provides strong indirect evidence and a physiological link to the clinical significance of ultrafiltration volume. TBW is a static indicator of volume load, whereas ultrafiltration volume is the core dynamic intervention for clinically adjusting TBW and achieving volume balance. Thus, the association between TBW and hypertension fundamentally underscores the core value of refined, individualized ultrafiltration management for blood pressure control.

In response to the above, we explicitly supplemented the following three points in the “Discussion” section (Limitations paragraph) of our paper: incomplete information on antihypertensive medications, lack of assessment for blood pressure variability, and failure to include ultrafiltration volume and other parameters in the analysis constitute the limitations of this study. Future studies will prospectively integrate these therapeutic parameters with body composition metrics to establish a comprehensive evidence chain spanning “therapeutic interventions” to “physiological states” and ultimately “clinical outcomes,” thereby providing a foundation for precision management. We sincerely appreciate your rigorous review of this study design; your insights have significantly enhanced its integrity and clinical relevance.

**2) Additionally, there are some inconsistencies in wording and some typographical errors which would benefit from careful revision to improve clarity and overall presentation.**

**Response:** Thanks for your valuable comments. We have conducted a comprehensive proofreading of the entire text for language and formatting, correcting inconsistent terminology, table formatting errors, and grammatical issues (e.g.: 1. Standardized “P-value” in tables to “P”; 2. Standardized “Lasso regression” to “LASSO regression”; 3. Typo in the fifth paragraph of the Discussion section: “Inaddition, there is a...” corrected to “In addition, there is a...”, etc.).
