# Supplementary material for: Relationship between hypertension during dialysis and body composition in non-overweight/overweight obese patients
Source: PLoS One. 2026 Apr 21;21(4):e0333566. doi: 10.1371/journal.pone.0333566 (PMC13098967; doi:10.1371/journal.pone.0333566)
Supplement: Attachment — Submitted filename: Response_to_Reviewers_auresp_2.docx [file pone.0333566.s004.docx]

**Additional Editor Comments:**

**Dear Authors,**

**Please revise the manuscript by carefully addressing the comments raised by the reviewer #3.**

**Response:**

Dear Editor,

Thank you for your guidance. We have carefully revised the manuscript according to **reviewer #3**'s comments, addressing each point raised and incorporating the corresponding modifications into the manuscript. The revisions are detailed in the response letter. We kindly request your re-review. Should any further modifications be required, we are ready to cooperate fully.

**[Note: HTML markup is below. Please do not edit.]**

**Reviewers' comments:**

**Reviewer's Responses to Questions**

**Comments to the Author**

**1. If the authors have adequately addressed your comments raised in a previous round of review and you feel that this manuscript is now acceptable for publication, you may indicate that here to bypass the “Comments to the Author” section, enter your conflict of interest statement in the “Confidential to Editor” section, and submit your "Accept" recommendation.**

**Reviewer #1: All comments have been addressed**

**Reviewer #3: All comments have been addressed**

**2. Is the manuscript technically sound, and do the data support the conclusions?**

**The manuscript must describe a technically sound piece of scientific research with data that supports the conclusions. Experiments must have been conducted rigorously, with appropriate controls, replication, and sample sizes. The conclusions must be drawn appropriately based on the data presented.**

**Reviewer #1: Yes**

**Reviewer #3: No**

**Response:** We appreciate the reviewer's attention. We have re-examined the data analysis process and interpretation of conclusions. This study employed LASSO regression for variable selection, combined with multivariate linear regression and logistic regression analyses stratified by gender and BMI. Results demonstrated significant correlations between specific body composition parameters (e.g., TBW, Protein, BMC, Fat) and hypertension, with statistical significance observed in specific subgroups. Despite limitations inherent to cross-sectional study designs, we believe the data provide reasonable support for our conclusions. We have further supplemented the discussion section with explanations regarding potential confounders (e.g., medication use, residual renal function) and emphasized the exploratory nature of this study, recommending future validation studies.

**3. Has the statistical analysis been performed appropriately and rigorously?**

**Reviewer #1: Yes**

**Reviewer #3: I Don't Know**

**Response:** We appreciate the reviewer's attention. We have supplemented the statistical methods section with more detailed explanations. (For example: LASSO regression was used for variable selection to avoid overfitting and multicollinearity, followed by multivariate regression analysis adjusted for age. All models underwent hypothesis testing, including linearity and residual normality.)

**4. Have the authors made all data underlying the findings in their manuscript fully available?**

**The PLOS Data policy requires authors to make all data underlying the findings described in their manuscript fully available without restriction, with rare exception (please refer to the Data Availability Statement in the manuscript PDF file). The data should be provided as part of the manuscript or its supporting information, or deposited to a public repository. For example, in addition to summary statistics, the data points behind means, medians and variance measures should be available. If there are restrictions on publicly sharing data—e.g. participant privacy or use of data from a third party—those must be specified.**

**Reviewer #1: Yes**

**Reviewer #3: No**

**Response:** We appreciate the reviewers' attention. In accordance with PLOS data policy, we have uploaded a de-identified dataset as supplementary material submitted alongside the revised manuscript to ensure data transparency while safeguarding patient privacy.

**5. Is the manuscript presented in an intelligible fashion and written in standard English?**

**PLOS ONE does not copyedit accepted manuscripts, so the language in submitted articles must be clear, correct, and unambiguous. Any typographical or grammatical errors should be corrected at revision, so please note any specific errors here.**

**Reviewer #1: Yes**

**Reviewer #3: No**

**Response:** We appreciate the reviewers' attention. We have conducted a thorough linguistic review of the entire manuscript and engaged a native English-speaking colleague with medical writing expertise to assist in the revision. This ensures clear and accurate language expression and compliance with PLOS ONE's language standards.

**6. Review Comments to the Author**

**Please use the space provided to explain your answers to the questions above. You may also include additional comments for the author, including concerns about dual publication, research ethics, or publication ethics. (Please upload your review as an attachment if it exceeds 20,000 characters)**

**Reviewer #1: (No Response)**

**Reviewer #3: Dear Authors,**

**Response: Thank you very much for submitting the manuscript entitled “Relationship between Hypertension During Dialysis and Body Composition in Non-Overweight/Overweight Obese Patients.”**

**This is an original study that aims to explore a possible association between intradialytic hypertension and body composition. I appreciate the effort made by the authors in addressing the comments raised by the previous reviewers.**

**However, several limitations may affect the robustness and clinical applicability of the findings, making the statistical analyses difficult to translate into routine clinical practice.**

**In particular, did the authors collect data regarding the menopausal status of the women included in the study or the use of ongoing oestrogen therapy? Oestrogen levels may influence blood pressure regulation and could therefore represent a relevant variable when interpreting sex-related differences. Including this information might help to better contextualise the observed findings.**

**Furthermore, can the authors hypothesise a potential explanation for the sex-related differences observed in the only statistically significant parameters identified?**

**Finally, it would be helpful if the authors could further clarify how these results should be interpreted in a clinical context and whether they may have practical implications for patient management.**

**Response:** Thanks for your valuable comments. First, we fully concur with the reviewer's perspective that menopausal status and estrogen use among female patients may significantly influence blood pressure regulation, thereby constituting potential confounding factors when interpreting gender-related differences. Unfortunately, our center's information system does not collect data on menopausal status or estrogen replacement therapy use among female patients. This represents a limitation of the study, which we have addressed in the section discussing study limitations. In future prospective studies, we will fully consider this critical recommendation by systematically collecting relevant data to more comprehensively explore the moderating role of sex hormones in the relationship between body composition and hypertension.

Secondly, we have added the following hypothesis at the end of the fifth paragraph in the Discussion section: Gender-specific mechanisms related to fat distribution patterns, sex hormone levels, and insulin resistance may account for the gender-related differences observed in this study. We hope to gain your acknowledgment and support for this point. (Content as follows: The gender-specific mechanisms underlying fat distribution patterns, sex hormone levels, and insulin resistance may account for sex-related differences. Sironi[45] et al. noted that visceral fat is closely associated with hypertension, insulin resistance, and metabolic syndrome. Postmenopausal women experience increased visceral fat accumulation due to declining estrogen levels, making adipose tissue a primary driver of hypertension. In male patients of this study, the association between TBW and Protein with hypertension may reflect more severe metabolic disorders and a state of protein-energy depletion. Conversely, the role of Fat in female patients more directly highlights the core position of adipose tissue as an endocrine organ in blood pressure regulation.)

Finally, we further elaborate on the clinical implications at the end of paragraph 6 in the Discussion section, hoping to gain your endorsement and support. (For example, in non-overweight males, monitoring total body water (TBW) and protein status aids in identifying hypertension risk; in overweight/obese females, controlling body fat may be more beneficial for blood pressure management. This suggests that individualized intervention strategies should be developed based on body composition rather than BMI alone.)

**7. PLOS authors have the option to publish the peer review history of their article (what does this mean?). If published, this will include your full peer review and any attached files.**

**If you choose “no”, your identity will remain anonymous but your review may still be made public.**

**Response: Yes**

**Do you want your identity to be public for this peer review? For information about this choice, including consent withdrawal, please see our Privacy Policy.**

**Reviewer #1: No**

**Reviewer #3: No**
