# Supplementary material for: Relationship between hypertension during dialysis and body composition in non-overweight/overweight obese patients
Source: PLoS One. 2026 Apr 21;21(4):e0333566. doi: 10.1371/journal.pone.0333566 (PMC13098967; doi:10.1371/journal.pone.0333566)
Supplement: Peer review history

# Peer review: Decision Letter 0

**DOI:** 10.1371/journal.pone.0333566.r001

**Authors:** Diego Moriconi

28 Dec 2025

Dear Dr. Wang,

Please submit your revised manuscript by Feb 11 2026 11:59PM. If you will need more time than this to complete your revisions, please reply to this message or contact the journal office at plosone@plos.org . When you're ready to submit your revision, log on to https://www.editorialmanager.com/pone/ and select the 'Submissions Needing Revision' folder to locate your manuscript file.. When you're ready to submit your revision, log on to https://www.editorialmanager.com/pone/ and select the 'Submissions Needing Revision' folder to locate your manuscript file.

- A letter that responds to each point raised by the academic editor and reviewer(s). You should upload this letter as a separate file labeled 'Response to Reviewers'.
- A marked-up copy of your manuscript that highlights changes made to the original version. You should upload this as a separate file labeled 'Revised Manuscript with Track Changes'.
- An unmarked version of your revised paper without tracked changes. You should upload this as a separate file labeled 'Manuscript'.

If you would like to make changes to your financial disclosure, please include your updated statement in your cover letter. Guidelines for resubmitting your figure files are available below the reviewer comments at the end of this letter.

If applicable, we recommend that you deposit your laboratory protocols in protocols.io to enhance the reproducibility of your results. Protocols.io assigns your protocol its own identifier (DOI) so that it can be cited independently in the future. For instructions see: https://journals.plos.org/plosone/s/submission-guidelines#loc-laboratory-protocols . Additionally, PLOS ONE offers an option for publishing peer-reviewed Lab Protocol articles, which describe protocols hosted on protocols.io. Read more information on sharing protocols at . Additionally, PLOS ONE offers an option for publishing peer-reviewed Lab Protocol articles, which describe protocols hosted on protocols.io. Read more information on sharing protocols at https://plos.org/protocols?utm_medium=editorial-email&utm_source=authorletters&utm_campaign=protocols ..

We look forward to receiving your revised manuscript.

Kind regards,

Diego Moriconi

Academic Editor

PLOS One

Journal Requirements:

When submitting your revision, we need you to address these additional requirements.

1. Please ensure that your manuscript meets PLOS ONE's style requirements, including those for file naming. The PLOS ONE style templates can be found at

https://journals.plos.org/plosone/s/file?id=wjVg/PLOSOne_formatting_sample_main_body.pdf and

https://journals.plos.org/plosone/s/file?id=ba62/PLOSOne_formatting_sample_title_authors_affiliations.pdf

2. PLOS requires an ORCID iD for the corresponding author in Editorial Manager on papers submitted after December 6th, 2016. Please ensure that you have an ORCID iD and that it is validated in Editorial Manager. To do this, go to ‘Update my Information’ (in the upper left-hand corner of the main menu), and click on the Fetch/Validate link next to the ORCID field. This will take you to the ORCID site and allow you to create a new iD or authenticate a pre-existing iD in Editorial Manager.

3. Thank you for stating the following financial disclosure:

“This study was supported by the Chongqing Science and Health Joint Medical Research Top Project [2025MSXM108]”

Please state what role the funders took in the study.  If the funders had no role, please state: "The funders had no role in study design, data collection and analysis, decision to publish, or preparation of the manuscript."

If this statement is not correct you must amend it as needed.

Please include this amended Role of Funder statement in your cover letter; we will change the online submission form on your behalf.

4. In this instance it seems there may be acceptable restrictions in place that prevent the public sharing of your minimal data. However, in line with our goal of ensuring long-term data availability to all interested researchers, PLOS’ Data Policy states that authors cannot be the sole named individuals responsible for ensuring data access (http://journals.plos.org/plosone/s/data-availability#loc-acceptable-data-sharing-methods).

Data requests to a non-author institutional point of contact, such as a data access or ethics committee, helps guarantee long term stability and availability of data. Providing interested researchers with a durable point of contact ensures data will be accessible even if an author changes email addresses, institutions, or becomes unavailable to answer requests.

Before we proceed with your manuscript, please also provide non-author contact information (phone/email/hyperlink) for a data access committee, ethics committee, or other institutional body to which data requests may be sent. If no institutional body is available to respond to requests for your minimal data, please consider if there any institutional representatives who did not collaborate in the study, and are not listed as authors on the manuscript, who would be able to hold the data and respond to external requests for data access? If so, please provide their contact information (i.e., email address). Please also provide details on how you will ensure persistent or long-term data storage and availability.

5. If the reviewer comments include a recommendation to cite specific previously published works, please review and evaluate these publications to determine whether they are relevant and should be cited. There is no requirement to cite these works unless the editor has indicated otherwise.

Additional Editor Comments:

Dear authors, thanking you for your work and for choosing this journal, I must unfortunately point out that the paper must be profoundly modified for it to be suitable for publication.

Reviewers' comments:

Reviewer's Responses to Questions

**Comments to the Author**

1. Is the manuscript technically sound, and do the data support the conclusions?

Reviewer #1: Yes

Reviewer #2: No

**********

2. Has the statistical analysis been performed appropriately and rigorously?

Reviewer #1: Yes

Reviewer #2: No

**********

3. Have the authors made all data underlying the findings in their manuscript fully available?

The PLOS Data policy requires authors to make all data underlying the findings described in their manuscript fully available without restriction, with rare exception (please refer to the Data Availability Statement in the manuscript PDF file). The data should be provided as part of the manuscript or its supporting information, or deposited to a public repository. For example, in addition to summary statistics, the data points behind means, medians and variance measures should be available. If there are restrictions on publicly sharing data—e.g. participant privacy or use of data from a third party—those must be specified.

Reviewer #1: Yes

Reviewer #2: Yes

**********

4. Is the manuscript presented in an intelligible fashion and written in standard English?

Reviewer #1: Yes

Reviewer #2: No

**********

Reviewer #1: Dear authors, congratulations on the manuscript and interest in the subject of hypertension in dialysis. I have a few comments I'd like to make about the work:

1) At what point during the hemodialysis session was the blood pressure value captured for the diagnosis of arterial hypertension?

2) How many individuals were excluded from the study based on the exclusion criteria?

3) Why was LASSO regression used and not ELASTIC NET regression ?

4) What about residual renal function? We know that loss of residual renal function compromises blood volume control and can impact blood pressure, while also contributing to changes in body composition. Could the men in this study, who had a correlation between total body water and systolic blood pressure, have lower residual renal function ?

5) The authors suggested that mineral and bone metabolism disorders could partially explain the correlation between bone mass composition parameters and arterial hypertension in men. However, in this study, this is merely speculative, since hypercalciuria appears to be negligible in dialysis patients, and the lack of laboratory data on mineral metabolism variables (parathyroid hormone, calcium, and phosphorus levels) precludes any conclusions in this regard. I agree, however, that new studies evaluating these laboratory variables should be carried out.

6) The question of "reverse epidemiology" in patients receiving dialysis is quite interesting, but it links obesity and overweight to lower mortality, and does not necessarily include hypertension in this conclusion. I would like the authors to weigh in more assertively at this point, specifically in dialysis patients.

7) Diabetic patients with chronic kidney disease have higher blood pressure values and are hypervolemic. Why was the variable diabetes mellitus not considered in this study?

Reviewer #2: Thank you for the effort and work put into this study. Hypertension is highly prevalent among MHD patients and is influenced by multiple inter-related factors. To further strengthen the findings and interpretation, additional information would be helpful, such as blood pressure variability across the dialysis phases (pre-, intra- and post-haemodialysis), details of antihypertensive medications and compliant, ultrafiltration volume, and other dialysis-related parameters as mentioned. These variables are important to better justify and contextualise the associations with BIA parameters. Incorporating these, may enhance the clinical relevance of the study and provide deeper insights into how BIA findings can be applied to optimise blood pressure management in MHD patients.

Additionally, there are some inconsistencies in wording and some typographical errors which would benefit from careful revision to improve clarity and overall presentation.

**********

what does this mean? ). If published, this will include your full peer review and any attached files.). If published, this will include your full peer review and any attached files.

If you choose “no”, your identity will remain anonymous but your review may still be made public.

**Do you want your identity to be public for this peer review?** For information about this choice, including consent withdrawal, please see our For information about this choice, including consent withdrawal, please see our Privacy Policy .

Reviewer #1: No

Reviewer #2: No

**********

[NOTE: If reviewer comments were submitted as an attachment file, they will be attached to this email and accessible via the submission site. Please log into your account, locate the manuscript record, and check for the action link "View Attachments". If this link does not appear, there are no attachment files.]

To ensure your figures meet our technical requirements, please review our figure guidelines: https://journals.plos.org/plosone/s/figures

You may also use PLOS’s free figure tool, NAAS, to help you prepare publication quality figures: https://journals.plos.org/plosone/s/figures#loc-tools-for-figure-preparation.

NAAS will assess whether your figures meet our technical requirements by comparing each figure against our figure specifications.

# Author comment: Author response to Decision Letter 1

**DOI:** 10.1371/journal.pone.0333566.r002

8 Jan 2026

Reviewer #1:

Dear authors, congratulations on the manuscript and interest in the subject of hypertension in dialysis. I have a few comments I'd like to make about the work:

1) At what point during the hemodialysis session was the blood pressure value captured for the diagnosis of arterial hypertension?

Response: Thanks for your valuable comments. The blood pressure data used in this study were derived from the average blood pressure recorded during dialysis sessions in the dialysis center information system, specifically the average blood pressure values from each dialysis session within the three months preceding enrollment. Blood pressure during each dialysis session was measured and recorded by two professional nurses. We did not distinguish specific time periods (pre-, intra-, or post-dialysis) but instead used the overall average value throughout the entire dialysis session as the basis for analysis. This approach partially reflects the overall blood pressure burden during the dialysis process. In subsequent studies, we may further refine the time periods to analyze blood pressure heterogeneity.

2) How many individuals were excluded from the study based on the exclusion criteria?

Response: Thanks for your valuable comments. As you have considered, to further enhance the transparency and reproducibility of the study, we have supplemented the “Materials and Methods” section with details on the number of cases excluded during the screening process and their primary reasons.

3) Why was LASSO regression used and not ELASTIC NET regression ?

Response: Thanks for your valuable comments. LASSO regression is suitable for automatic variable selection in high-dimensional data, effectively preventing overfitting. It is appropriate for this study's initial scenario with a large number of variables (14). As you considered, Elastic Net combines the advantages of LASSO and ridge regression. However, when the sample size is moderate and multicollinearity among variables is not pronounced (as in this study), LASSO already performs adequate feature selection. Therefore, LASSO regression was selected.

4) What about residual renal function? We know that loss of residual renal function compromises blood volume control and can impact blood pressure, while also contributing to changes in body composition. Could the men in this study, who had a correlation between total body water and systolic blood pressure, have lower residual renal function?

Response: Thanks for your valuable comments and the question you raise is indeed critical. Residual kidney function (RKF) may indeed influence a patient's volume status and blood pressure levels by affecting the efficiency of sodium and water clearance. Although this study included only end-stage renal disease patients on regular dialysis for ≥3 months (most with severely impaired or absent RKF), inter-individual variations in residual renal function may still represent a potential confounding factor. This represents one limitation of our study, which we addressed in the Discussion section. Future research plans include incorporating more direct measures of RKF (such as urea clearance or urine output) to further elucidate its role in the relationship between body composition and blood pressure.

5) The authors suggested that mineral and bone metabolism disorders could partially explain the correlation between bone mass composition parameters and arterial hypertension in men. However, in this study, this is merely speculative, since hypercalciuria appears to be negligible in dialysis patients, and the lack of laboratory data on mineral metabolism variables (parathyroid hormone, calcium, and phosphorus levels) precludes any conclusions in this regard. I agree, however, that new studies evaluating these laboratory variables should be carried out.

Response: Thanks for your valuable comments and we fully agree with your perspective. The discussion regarding the association between bone mineral content and hypertension in the manuscript is indeed speculative. In future studies, we will incorporate additional laboratory indicators such as serum calcium, serum phosphorus, and parathyroid hormone to more comprehensively explore the relationship between mineral metabolism and blood pressure. We have revised the relevant discussion to ensure the rigor of our findings, as detailed at the end of the fourth paragraph in the Discussion section.

6) The question of "reverse epidemiology" in patients receiving dialysis is quite interesting, but it links obesity and overweight to lower mortality, and does not necessarily include hypertension in this conclusion. I would like the authors to weigh in more assertively at this point, specifically in dialysis patients.

Response: Thanks for your valuable comments. In the sixth paragraph under discussion, we supplement and emphasize that: although an “inverse epidemiological” phenomenon linking obesity to mortality may exist in the dialysis population, this does not negate obesity's role as a risk factor for hypertension. Our study provides new evidence for understanding the complex relationship between obesity and hypertension in dialysis patients from the perspective of body composition analysis. It further underscores that actively managing hypertension driven by factors such as body fat remains crucial in the clinical management of hemodialysis patients, even for those who may benefit from a higher BMI in terms of survival.

7) Diabetic patients with chronic kidney disease have higher blood pressure values and are hypervolemic. Why was the variable diabetes mellitus not considered in this study?

Response: Thanks for your valuable comments. As you have noted, diabetes is a common and significant comorbidity among patients on maintenance hemodialysis, with profound effects on blood pressure, volume load, and metabolic status. During the study design phase, to more clearly examine the direct association between body composition and hypertension while avoiding strong confounding effects from diabetes and its related complications (such as autonomic neuropathy and more complex volume issues), diabetic patients were deliberately excluded. Therefore, the conclusions of this study primarily apply to the non-diabetic MHD population. We have supplemented this point in the limitations section of the “Discussion” and emphasize that future research should validate these associations in diabetic populations to provide more universally applicable clinical guidance.

Reviewer #2:

1) Thank you for the effort and work put into this study. Hypertension is highly prevalent among MHD patients and is influenced by multiple inter-related factors. To further strengthen the findings and interpretation, additional information would be helpful, such as blood pressure variability across the dialysis phases (pre-, intra- and post-haemodialysis), details of antihypertensive medications and compliant, ultrafiltration volume, and other dialysis-related parameters as mentioned. These variables are important to better justify and contextualise the associations with BIA parameters. Incorporating these, may enhance the clinical relevance of the study and provide deeper insights into how BIA findings can be applied to optimise blood pressure management in MHD patients.

Response: Thanks for your valuable comments. We fully agree that parameters such as blood pressure variability, antihypertensive medication use, and ultrafiltration volume are crucial for gaining a deeper understanding of the relationship between body composition and blood pressure in the context of hemodialysis. Due to the retrospective design of this study, there were certain limitations in obtaining some parameters:

Antihypertensive Medications: Patients used a wide variety of antihypertensive drugs. As this was a retrospective analysis, systematic classification and dose adjustment analysis (e.g., specific types, doses, and medication adherence) were challenging. Therefore, these were not included as covariates in the model. We further acknowledge this as a limitation of the study.

Blood pressure variability: To minimize random error from single measurements, we used the average of all blood pressure readings taken during dialysis sessions within the three months prior to enrollment to reflect the patient's overall blood pressure burden. However, this approach cannot precisely quantify dynamic blood pressure changes during dialysis (e.g., blood pressure variability or intradialytic hypotension events). We have supplemented this point in the discussion as a direction for future research.

Ultrafiltration Volume: Your point is crucial. While this study did not directly include ultrafiltration volume in the model, one of its core findings—that total body water (TBW) is an independent positive predictor of hypertension in non-overweight male patients—provides strong indirect evidence and a physiological link to the clinical significance of ultrafiltration volume. TBW is a static indicator of volume load, whereas ultrafiltration volume is the core dynamic intervention for clinically adjusting TBW and achieving volume balance. Thus, the association between TBW and hypertension fundamentally underscores the core value of refined, individualized ultrafiltration management for blood pressure control.

In response to the above, we explicitly supplemented the following three points in the “Discussion” section (Limitations paragraph) of our paper: incomplete information on antihypertensive medications, lack of assessment for blood pressure variability, and failure to include ultrafiltration volume and other parameters in the analysis constitute the limitations of this study. Future studies will prospectively integrate these therapeutic parameters with body composition metrics to establish a comprehensive evidence chain spanning “therapeutic interventions” to “physiological states” and ultimately “clinical outcomes,” thereby providing a foundation for precision management. We sincerely appreciate your rigorous review of this study design; your insights have significantly enhanced its integrity and clinical relevance.

2) Additionally, there are some inconsistencies in wording and some typographical errors which would benefit from careful revision to improve clarity and overall presentation.

Response: Thanks for your valuable comments. We have conducted a comprehensive proofreading of the entire text for language and formatting, correcting inconsistent terminology, table formatting errors, and grammatical issues (e.g.: 1. Standardized “P-value” in tables to “P”; 2. Standardized “Lasso regression” to “LASSO regression”; 3. Typo in the fifth paragraph of the Discussion section: “Inaddition, there is a...” corrected to “In addition, there is a...”, etc.).

Attachment Submitted filename: Response to Reviewers.docx

# Peer review: Decision Letter 1

**DOI:** 10.1371/journal.pone.0333566.r003

**Authors:** Diego Moriconi

4 Mar 2026

Dear Dr. Yunyan Wang,

Thank you for submitting your manuscript to PLOS ONE. After careful consideration, we feel that it has merit but does not fully meet PLOS ONE’s publication criteria as it currently stands. Therefore, we invite you to submit a revised version of the manuscript that addresses the points raised during the review process.

Please submit your revised manuscript by  Apr 18 2026 11:59PM. If you will need more time than this to complete your revisions, please reply to this message or contact the journal office at plosone@plos.org . When you're ready to submit your revision, log on to https://www.editorialmanager.com/pone/ and select the 'Submissions Needing Revision' folder to locate your manuscript file.. When you're ready to submit your revision, log on to https://www.editorialmanager.com/pone/ and select the 'Submissions Needing Revision' folder to locate your manuscript file.

- A letter that responds to each point raised by the academic editor and reviewer(s). You should upload this letter as a separate file labeled 'Response to Reviewers'.
- A marked-up copy of your manuscript that highlights changes made to the original version. You should upload this as a separate file labeled 'Revised Manuscript with Track Changes'.
- An unmarked version of your revised paper without tracked changes. You should upload this as a separate file labeled 'Manuscript'.

If you would like to make changes to your financial disclosure, please include your updated statement in your cover letter. Guidelines for resubmitting your figure files are available below the reviewer comments at the end of this letter.

If applicable, we recommend that you deposit your laboratory protocols in protocols.io to enhance the reproducibility of your results. Protocols.io assigns your protocol its own identifier (DOI) so that it can be cited independently in the future. For instructions see: https://journals.plos.org/plosone/s/submission-guidelines#loc-laboratory-protocols . Additionally, PLOS ONE offers an option for publishing peer-reviewed Lab Protocol articles, which describe protocols hosted on protocols.io. Read more information on sharing protocols at . Additionally, PLOS ONE offers an option for publishing peer-reviewed Lab Protocol articles, which describe protocols hosted on protocols.io. Read more information on sharing protocols at https://plos.org/protocols?utm_medium=editorial-email&utm_source=authorletters&utm_campaign=protocols ..

We look forward to receiving your revised manuscript.

Kind regards,

Diego Moriconi

Academic Editor

PLOS One

Journal Requirements:

If the reviewer comments include a recommendation to cite specific previously published works, please review and evaluate these publications to determine whether they are relevant and should be cited. There is no requirement to cite these works unless the editor has indicated otherwise.

Additional Editor Comments:

Dear Authors,

Please revise the manuscript by carefully addressing the comments raised by the reviewer #3.

[Note: HTML markup is below. Please do not edit.]

Reviewers' comments:

Reviewer's Responses to Questions

**Comments to the Author**

Reviewer #1: All comments have been addressed

Reviewer #3: All comments have been addressed

**********

2. Is the manuscript technically sound, and do the data support the conclusions?

Reviewer #1: Yes

Reviewer #3: No

**********

3. Has the statistical analysis been performed appropriately and rigorously?

Reviewer #1: Yes

Reviewer #3: I Don't Know

**********

4. Have the authors made all data underlying the findings in their manuscript fully available?

The PLOS Data policy requires authors to make all data underlying the findings described in their manuscript fully available without restriction, with rare exception (please refer to the Data Availability Statement in the manuscript PDF file). The data should be provided as part of the manuscript or its supporting information, or deposited to a public repository. For example, in addition to summary statistics, the data points behind means, medians and variance measures should be available. If there are restrictions on publicly sharing data—e.g. participant privacy or use of data from a third party—those must be specified.

Reviewer #1: Yes

Reviewer #3: No

**********

5. Is the manuscript presented in an intelligible fashion and written in standard English?

Reviewer #1: Yes

Reviewer #3: No

**********

Reviewer #1: (No Response)

Reviewer #3: Dear Authors,

Thank you very much for submitting the manuscript entitled “Relationship between Hypertension During Dialysis and Body Composition in Non-Overweight/Overweight Obese Patients.”

This is an original study that aims to explore a possible association between intradialytic hypertension and body composition. I appreciate the effort made by the authors in addressing the comments raised by the previous reviewers.

However, several limitations may affect the robustness and clinical applicability of the findings, making the statistical analyses difficult to translate into routine clinical practice.

In particular, did the authors collect data regarding the menopausal status of the women included in the study or the use of ongoing oestrogen therapy? Oestrogen levels may influence blood pressure regulation and could therefore represent a relevant variable when interpreting sex-related differences. Including this information might help to better contextualise the observed findings.

Furthermore, can the authors hypothesise a potential explanation for the sex-related differences observed in the only statistically significant parameters identified?

Finally, it would be helpful if the authors could further clarify how these results should be interpreted in a clinical context and whether they may have practical implications for patient management.

**********

what does this mean? ). If published, this will include your full peer review and any attached files.). If published, this will include your full peer review and any attached files.

If you choose “no”, your identity will remain anonymous but your review may still be made public.

**Do you want your identity to be public for this peer review?** For information about this choice, including consent withdrawal, please see our For information about this choice, including consent withdrawal, please see our Privacy Policy .

Reviewer #1: No

Reviewer #3: No

**********

[NOTE: If reviewer comments were submitted as an attachment file, they will be attached to this email and accessible via the submission site. Please log into your account, locate the manuscript record, and check for the action link "View Attachments". If this link does not appear, there are no attachment files.]

To ensure your figures meet our technical requirements, please review our figure guidelines: https://journals.plos.org/plosone/s/figures

You may also use PLOS’s free figure tool, NAAS, to help you prepare publication quality figures: https://journals.plos.org/plosone/s/figures#loc-tools-for-figure-preparation.

NAAS will assess whether your figures meet our technical requirements by comparing each figure against our figure specifications.

# Author comment: Author response to Decision Letter 2

**DOI:** 10.1371/journal.pone.0333566.r004

13 Mar 2026

Additional Editor Comments:

Dear Authors,

Please revise the manuscript by carefully addressing the comments raised by the reviewer #3.

Response:

Dear Editor,

Thank you for your guidance. We have carefully revised the manuscript according to reviewer #3's comments, addressing each point raised and incorporating the corresponding modifications into the manuscript. The revisions are detailed in the response letter. We kindly request your re-review. Should any further modifications be required, we are ready to cooperate fully.

[Note: HTML markup is below. Please do not edit.]

Reviewers' comments:

Reviewer's Responses to Questions

Comments to the Author

1. If the authors have adequately addressed your comments raised in a previous round of review and you feel that this manuscript is now acceptable for publication, you may indicate that here to bypass the “Comments to the Author” section, enter your conflict of interest statement in the “Confidential to Editor” section, and submit your "Accept" recommendation.

Reviewer #1: All comments have been addressed

Reviewer #3: All comments have been addressed

2. Is the manuscript technically sound, and do the data support the conclusions?

The manuscript must describe a technically sound piece of scientific research with data that supports the conclusions. Experiments must have been conducted rigorously, with appropriate controls, replication, and sample sizes. The conclusions must be drawn appropriately based on the data presented.

Reviewer #1: Yes

Reviewer #3: No

Response: We appreciate the reviewer's attention. We have re-examined the data analysis process and interpretation of conclusions. This study employed LASSO regression for variable selection, combined with multivariate linear regression and logistic regression analyses stratified by gender and BMI. Results demonstrated significant correlations between specific body composition parameters (e.g., TBW, Protein, BMC, Fat) and hypertension, with statistical significance observed in specific subgroups. Despite limitations inherent to cross-sectional study designs, we believe the data provide reasonable support for our conclusions. We have further supplemented the discussion section with explanations regarding potential confounders (e.g., medication use, residual renal function) and emphasized the exploratory nature of this study, recommending future validation studies.

3. Has the statistical analysis been performed appropriately and rigorously?

Reviewer #1: Yes

Reviewer #3: I Don't Know

Response: We appreciate the reviewer's attention. We have supplemented the statistical methods section with more detailed explanations. (For example: LASSO regression was used for variable selection to avoid overfitting and multicollinearity, followed by multivariate regression analysis adjusted for age. All models underwent hypothesis testing, including linearity and residual normality.)

4. Have the authors made all data underlying the findings in their manuscript fully available?

The PLOS Data policy requires authors to make all data underlying the findings described in their manuscript fully available without restriction, with rare exception (please refer to the Data Availability Statement in the manuscript PDF file). The data should be provided as part of the manuscript or its supporting information, or deposited to a public repository. For example, in addition to summary statistics, the data points behind means, medians and variance measures should be available. If there are restrictions on publicly sharing data—e.g. participant privacy or use of data from a third party—those must be specified.

Reviewer #1: Yes

Reviewer #3: No

Response: We appreciate the reviewers' attention. In accordance with PLOS data policy, we have uploaded a de-identified dataset as supplementary material submitted alongside the revised manuscript to ensure data transparency while safeguarding patient privacy.

5. Is the manuscript presented in an intelligible fashion and written in standard English?

PLOS ONE does not copyedit accepted manuscripts, so the language in submitted articles must be clear, correct, and unambiguous. Any typographical or grammatical errors should be corrected at revision, so please note any specific errors here.

Reviewer #1: Yes

Reviewer #3: No

Response: We appreciate the reviewers' attention. We have conducted a thorough linguistic review of the entire manuscript and engaged a native English-speaking colleague with medical writing expertise to assist in the revision. This ensures clear and accurate language expression and compliance with PLOS ONE's language standards.

6. Review Comments to the Author

Please use the space provided to explain your answers to the questions above. You may also include additional comments for the author, including concerns about dual publication, research ethics, or publication ethics. (Please upload your review as an attachment if it exceeds 20,000 characters)

Reviewer #1: (No Response)

Reviewer #3: Dear Authors,

Response: Thank you very much for submitting the manuscript entitled “Relationship between Hypertension During Dialysis and Body Composition in Non-Overweight/Overweight Obese Patients.”

This is an original study that aims to explore a possible association between intradialytic hypertension and body composition. I appreciate the effort made by the authors in addressing the comments raised by the previous reviewers.

However, several limitations may affect the robustness and clinical applicability of the findings, making the statistical analyses difficult to translate into routine clinical practice.

In particular, did the authors collect data regarding the menopausal status of the women included in the study or the use of ongoing oestrogen therapy? Oestrogen levels may influence blood pressure regulation and could therefore represent a relevant variable when interpreting sex-related differences. Including this information might help to better contextualise the observed findings.

Furthermore, can the authors hypothesise a potential explanation for the sex-related differences observed in the only statistically significant parameters identified?

Finally, it would be helpful if the authors could further clarify how these results should be interpreted in a clinical context and whether they may have practical implications for patient management.

Response: Thanks for your valuable comments. First, we fully concur with the reviewer's perspective that menopausal status and estrogen use among female patients may significantly influence blood pressure regulation, thereby constituting potential confounding factors when interpreting gender-related differences. Unfortunately, our center's information system does not collect data on menopausal status or estrogen replacement therapy use among female patients. This represents a limitation of the study, which we have addressed in the section discussing study limitations. In future prospective studies, we will fully consider this critical recommendation by systematically collecting relevant data to more comprehensively explore the moderating role of sex hormones in the relationship between body composition and hypertension.

Secondly, we have added the following hypothesis at the end of the fifth paragraph in the Discussion section: Gender-specific mechanisms related to fat distribution patterns, sex hormone levels, and insulin resistance may account for the gender-related differences observed in this study. We hope to gain your acknowledgment and support for this point. (Content as follows: The gender-specific mechanisms underlying fat distribution patterns, sex hormone levels, and insulin resistance may account for sex-related differences. Sironi[45] et al. noted that visceral fat is closely associated with hypertension, insulin resistance, and metabolic syndrome. Postmenopausal women experience increased visceral fat accumulation due to declining estrogen levels, making adipose tissue a primary driver of hypertension. In male patients of this study, the association between TBW and Protein with hypertension may reflect more severe metabolic disorders and a state of protein-energy depletion. Conversely, the role of Fat in female patients more directly highlights the core position of adipose tissue as an endocrine organ in blood pressure regulation.)

Finally, we further elaborate on the clinical implications at the end of paragraph 6 in the Discussion section, hoping to gain your endorsement and support. (For example, in non-overweight males, monitoring total body water (TBW) and protein status aids in identifying hypertension risk; in overweight/obese females, controlling body fat may be more beneficial for blood pressure management. This suggests that individualized intervention strategies should be developed based on body composition rather than BMI alone.)

7. PLOS authors have the option to publish the peer review history of their article (what does this mean?). If published, this will include your full peer review and any attached files.

If you choose “no”, your identity will remain anonymous but your review may still be made public.

Response: Yes

Attachment Submitted filename: Response_to_Reviewers_auresp_2.docx

# Peer review: Decision Letter 2

**DOI:** 10.1371/journal.pone.0333566.r005

**Authors:** Diego Moriconi

5 Apr 2026

Relationship between Hypertension During Dialysis and Body Composition in Non-Overweight/Overweight Obese Patients

PONE-D-25-49455R2

Dear Dr. Yunyan Wang

We’re pleased to inform you that your manuscript has been judged scientifically suitable for publication and will be formally accepted for publication once it meets all outstanding technical requirements.

Within one week, you’ll receive an e-mail detailing the required amendments. When these have been addressed, you’ll receive a formal acceptance letter and your manuscript will be scheduled for publication.

An invoice will be generated when your article is formally accepted. Please note, if your institution has a publishing partnership with PLOS and your article meets the relevant criteria, all or part of your publication costs will be covered. Please make sure your user information is up-to-date by logging into Editorial Manager at Editorial Manager®  and clicking the ‘Update My Information' link at the top of the page. For questions related to billing, please contact  and clicking the ‘Update My Information' link at the top of the page. For questions related to billing, please contact billing support ..

If your institution or institutions have a press office, please notify them about your upcoming paper to help maximize its impact. If they’ll be preparing press materials, please inform our press team as soon as possible -- no later than 48 hours after receiving the formal acceptance. Your manuscript will remain under strict press embargo until 2 pm Eastern Time on the date of publication. For more information, please contact onepress@plos.org.

Kind regards,

Diego Moriconi

Academic Editor

PLOS One

Additional Editor Comments (optional):

Dear authors, after the changes made following the instructions of the reviewers, I communicate that the paper is suitable for publication

Reviewers' comments:

Reviewer's Responses to Questions

**Comments to the Author**

Reviewer #3: All comments have been addressed

**********

2. Is the manuscript technically sound, and do the data support the conclusions?

Reviewer #3: Yes

**********

3. Has the statistical analysis been performed appropriately and rigorously?

Reviewer #3: Yes

**********

4. Have the authors made all data underlying the findings in their manuscript fully available?

The PLOS Data policy requires authors to make all data underlying the findings described in their manuscript fully available without restriction, with rare exception (please refer to the Data Availability Statement in the manuscript PDF file). The data should be provided as part of the manuscript or its supporting information, or deposited to a public repository. For example, in addition to summary statistics, the data points behind means, medians and variance measures should be available. If there are restrictions on publicly sharing data—e.g. participant privacy or use of data from a third party—those must be specified.

Reviewer #3: Yes

**********

5. Is the manuscript presented in an intelligible fashion and written in standard English?

Reviewer #3: Yes

**********

Reviewer #3: Dear Authors,

Thank you very much for submitting the revised manuscript. All the questions have been answered properly, therefore in my opinion it is suitable for publication in PlosOne.

Best regards.

**********

what does this mean? ). If published, this will include your full peer review and any attached files.). If published, this will include your full peer review and any attached files.

If you choose “no”, your identity will remain anonymous but your review may still be made public.

**Do you want your identity to be public for this peer review?** For information about this choice, including consent withdrawal, please see our For information about this choice, including consent withdrawal, please see our Privacy Policy .

Reviewer #3: No

**********

# Editor report: Acceptance letter

**DOI:** 10.1371/journal.pone.0333566.r006

**Authors:** Diego Moriconi

PONE-D-25-49455R2

PLOS One

Dear Dr. Wang,

I'm pleased to inform you that your manuscript has been deemed suitable for publication in PLOS One. Congratulations! Your manuscript is now being handed over to our production team.

At this stage, our production department will prepare your paper for publication. This includes ensuring the following:

* All references, tables, and figures are properly cited

* All relevant supporting information is included in the manuscript submission,

* There are no issues that prevent the paper from being properly typeset

You will receive further instructions from the production team, including instructions on how to review your proof when it is ready. Please keep in mind that we are working through a large volume of accepted articles, so please give us a few days to review your paper and let you know the next and final steps.

Lastly, if your institution or institutions have a press office, please let them know about your upcoming paper now to help maximize its impact. If they'll be preparing press materials, please inform our press team within the next 48 hours. Your manuscript will remain under strict press embargo until 2 pm Eastern Time on the date of publication. For more information, please contact onepress@plos.org.

You will receive an invoice from PLOS for your publication fee after your manuscript has reached the completed accept phase. If you receive an email requesting payment before acceptance or for any other service, this may be a phishing scheme. Learn how to identify phishing emails and protect your accounts at https://explore.plos.org/phishing.

If we can help with anything else, please email us at customercare@plos.org.

Thank you for submitting your work to PLOS ONE and supporting open access.

Kind regards,

PLOS ONE Editorial Office Staff

on behalf of

Dr. Diego Moriconi

Academic Editor

PLOS One
